# Designing Tailored Molecular Cavities Using Calix[5]arenes as Building Blocks

**DOI:** 10.1002/tcr.202500281

**Published:** 2026-03-26

**Authors:** Takehiro Hirao, Takeharu Haino

**Affiliations:** ^1^ Department of Chemistry Graduate School of Advanced Science and Engineering Hiroshima University Higashi‐Hiroshima Japan; ^2^ International Institute for Sustainability with Knotted Chiral Meta Matter (SKCM2) Hiroshima University Higashi‐Hiroshima Japan

**Keywords:** calixarene, fullerene supramolecular polymer, host–guest chemistry, supramolecular chemistry

## Abstract

This study examines the shapes and dimensions of cavities defined by well‐designed molecular structures. We discuss our work on creating custom cavities using calix[5]arenes. The shapes and dimensions of these cavities are precisely determined to selectively interact with specific guest molecules. We demonstrate that strategic design leads to strong binding, guest selectivity, and responsiveness to stimuli. These results highlight the importance of cavity customization in advancing host–guest systems for potential applications in molecular recognition and supramolecular materials.

## Graphical abstract

This account focuses on the shape and dimensions of cavities formed by precisely designed molecular frameworks. It highlights our efforts to construct synthetically tailored cavities based on calix[5]arenes.

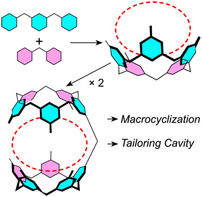

## 1 Introduction

**FIGURE 1:**
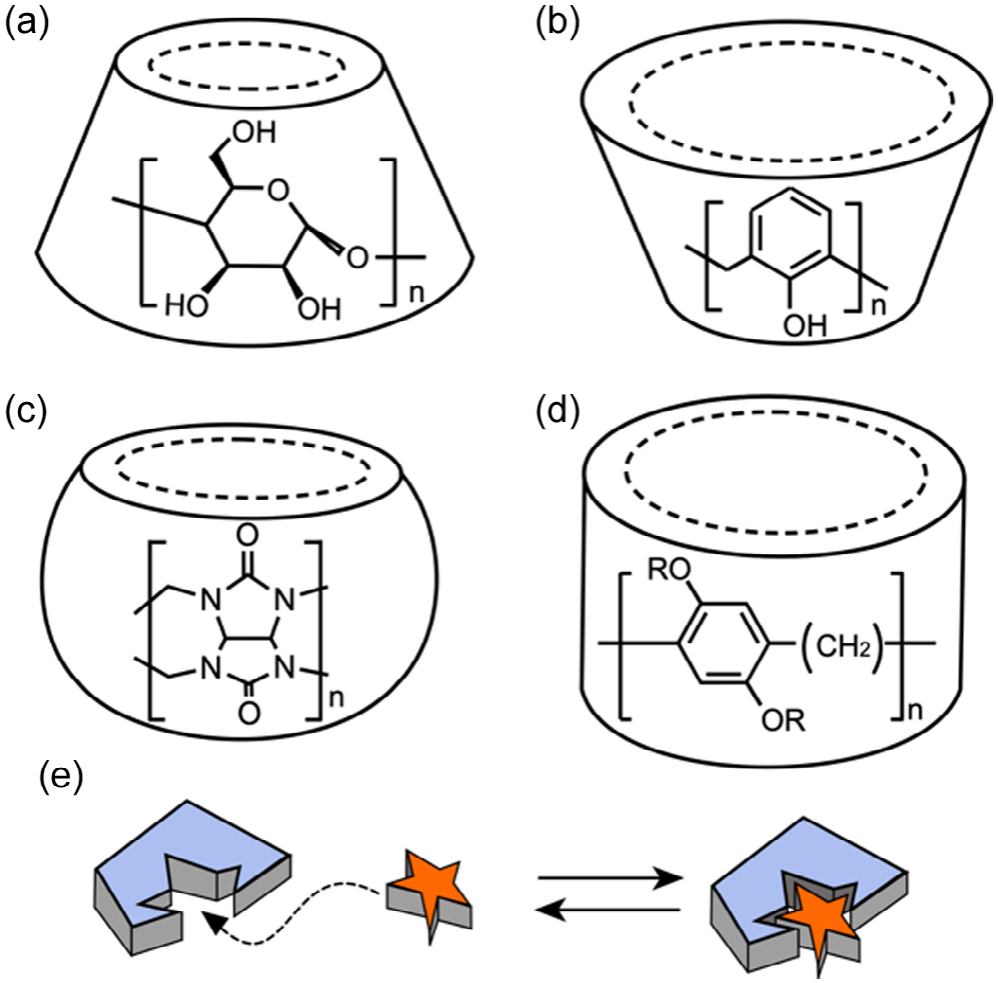
Cartoon representation of selected macrocyclic host molecules that have advanced the field of host–guest chemistry: (a) cyclodextrin, (b) calix[n]arene, (c) cucurbit[n]uril, and (d) pillar[n]arene. (e) Schematic cartoon of host–guest complexation, where a host molecule encapsulates a star‐shaped guest molecule in a half‐star‐shaped cavity to form the complex.

The molecular cavity is typically defined by the macrocyclic structures (Figure 1a) [1, 2, 3, 4, 5, 6, 7, 8, 9, 10, 11, 12, 13, 14]. Molecules with cavities can encapsulate smaller molecules within them to create host–guest complexes via non‐covalent intermolecular interactions, such as hydrogen bonding, electrostatic interactions, *π*–*π* stacking, van der Waals interactions, and charge–transfer interactions (Figure 1b). Synthetically tailored cavities refer to cavities in synthetic molecules designed to selectively encapsulate specific guest molecules. This customization alters host–guest complementarity [15, 16], enabling the creation of host molecules with shapes and dimensions suitable for the target guest molecules.

**FIGURE 2:**
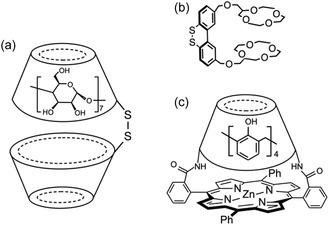
Cartoon representation of synthetically tailored cavities found between macrocyclic molecules: (a) bis(*β*‐cyclodextrin), (b) bis(crown ether), and (c) calix[4]arene‐capped porphyrin.

One of the most promising methods for creating newly shaped and dimensional cavities involves the use of macrocyclic molecules [17, 18, 19, 20, 21, 22, 23, 24, 25]. These custom‐made cavities demonstrate unique selectivity for encapsulating guests, surpassing that of individual macrocycles. For example, a covalently linked bis(*β*‐cyclodextrin) host displayed strong guest binding with an association constant exceeding 2.0 × 10^6^ mol L^–1^ for ethyl orange (Figure 2a) [26]. This notably high host–guest affinity, compared with *β*‐cyclodextrin, stems from the enlarged cavity formed by the two *β*‐cyclodextrin units that snugly accommodate the guest molecules. Covalently linked bis(crown ether) is a good example of synthetically tailored cavities. The two crown moieties are tethered using a disulfide‐bridged biphenyl linker to form a tweezer‐shaped host molecule (Figure 2b) [27]. The cavity between the two crown ethers is well‐structured when the linker is bridged by a disulfide bond. However, upon cleavage of the disulfide bond, the host loses its intercrown cavity due to the free rotation of molecular bonds. Consequently, the guest‐binding affinity of the bis(crown ether) host is significantly influenced by the presence or absence of disulfide bonds. A strategy involving cavity capping can yield structurally well‐defined synthetic cavities. Calix[4]arene‐capped Zn(II) porphyrins adopt a molecular configuration where the calix[4]arene cavity is enclosed by a porphyrin unit, creating a rigid cavity capable of recognizing aromatic guest molecules with minimal structural variances (Figure 2c) [28]. This host–guest complexation demonstrates high shape selectivity, a feature not observed in the individual components, namely calix[4]arene and porphyrin.

To date, several custom‐designed cavities have been created using newly developed macrocyclic molecules [29, 30, 31, 32, 33, 34, 35] and tweezer‐shaped molecules [36, 37, 38, 39, 40]. The advancement of host–guest chemistry has been driven by the customization of unique cavities, primarily defined by macrocyclic or tweezer‐shaped molecular structures. Our group has contributed to the field of host–guest chemistry using calix[4]arene‐ [41, 42, 43, 44], resorcinarene‐ [45, 46, 47, 48, 49, 50, 51, 52], bis(porphyrin)‐ [53, 54, 55, 56, 57, 58, 59, 60], and calix[5]arene‐ [61, 62, 63, 64] based host molecules. Among our host molecules, in this account, the calix[5]arene‐based host molecules are articulated. The aim was to provide a representative overview instead of a comprehensive listing of all research conducted in the field of host–guest chemistry. Accordingly, in the following section, we focus on calix[5]arene‐based host molecules, which are examples of cavities defined by macrocyclic and tweezer‐like structures. This account focuses on synthetically tailored cavities; thus, a synthetic scheme is briefly summarized to describe the strategy employed in their synthesis. Finally, we discuss our thoughts on the host–guest chemistry field.

## 2 Calix[5]arene‐Based Hosts

**FIGURE 3:**
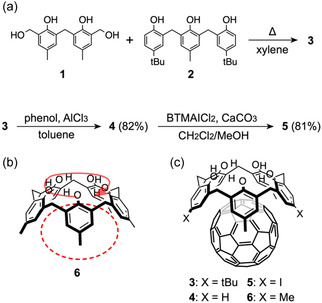
(a) Synthesis of calix[5]arene derivatives **3**–**5**. (b) Calix[5]arene **6** illustrates its cavity defined by cyclic hydrogen bonding. (c) Molecular structure of calix[5]arene derivatives **3**–**6** and [60]fullerene encapsulation.

We discovered that the bowl‐shaped cavity of calix[5]arenes is highly complementary to the structure of fullerenes, facilitating their selective encapsulation and the formation of stable host–guest complexes [65]. Calix[5]arenes are macrocyclic compounds comprising five para‐substituted phenolic moieties linked via a secondary carbon atom. The synthesis of calix[5]arenes involves the cyclization of methylene‐bridged substituted phenol dimers and trimers, such as **5** and **6**, in xylene [66, 67] or tetralin [68] (Figure 3a). The tert‐butyl groups at the para‐position can be eliminated through retro Friedel–Crafts alkylation to yield **3**. Subsequent iodization at the nonsubstituted para‐position of **3** using benzyltrimethylammonium dichloroiodate (BTMAICl_2_) produced **4**. The cyclic hydrogen bonding of the five phenolic hydroxyl groups forces the calix[5]arenes to adopt a bowl‐shaped conformation, creating a well‐defined large cavity (Figure 3b). The shape and dimensions of the cavity align well with the exterior of the fullerenes, resulting in a high binding affinity between them (Figure 3c). As summarized in Table 1, the binding constants between calix[5]arenes **4**–**6** and [60]fullerene have been reported to be approximately 500–2000 L mol^–1^, whereas binding constants of approximately 300–500 L mol^–1^ were estimated for calix[5]arenes **4**–**6** and [70]fullerene. This indicates that the shape and dimensions of the cavity defined by the calix[5]arene provide a better fit for [60]fullerene than for [70]fullerene. Considering that a calix[5]arene covers only one side of a fullerene sphere, a rational molecular design is required to maximize the intermolecular interactions to construct more stable host–guest complexes.

**TABLE 1:** Summary of binding constants (*K*_a_/L mol^–1^) between calix[5]arenes **4–6** and [60]/[70]fullerenes in toluene.

	C_60_	C_70_
**4**	590 ± 70	310 ± 30
**5**	2,100 ± 100	520 ± 60
**6**	1,670 ± 70	380 ± 30

**FIGURE 4:**
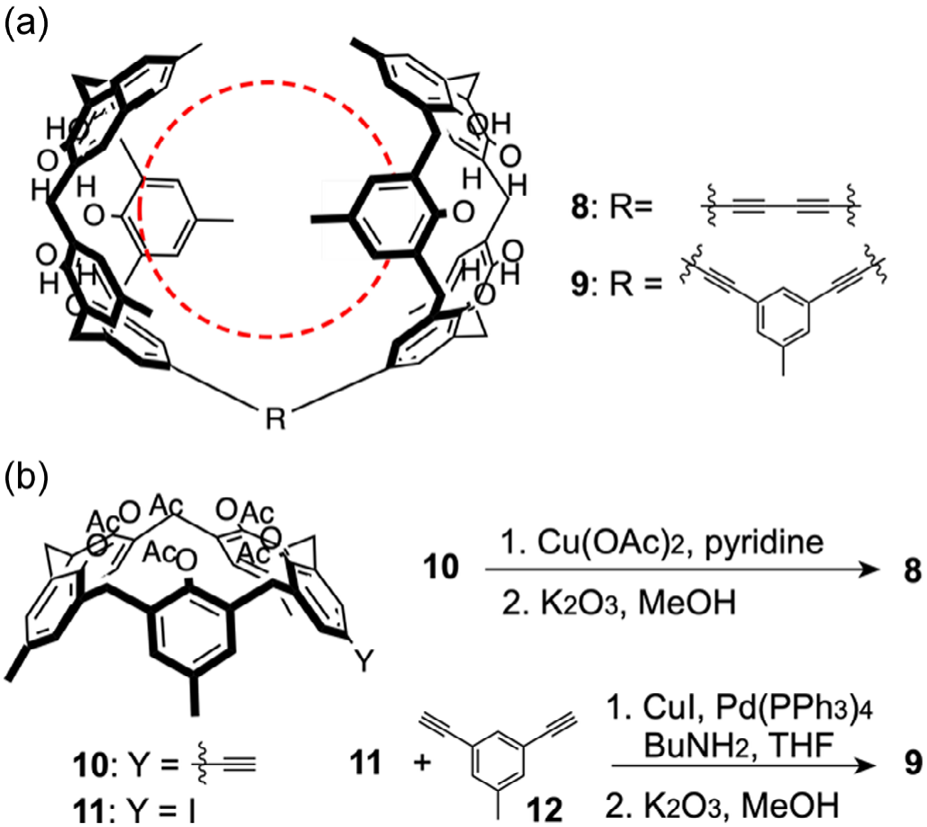
(a) Molecular structure of biscalix[5]arenes **8**,**9** illustrating the cavity formed between two calix[5]arene moieties. (b) Synthesis of biscalix[5]arenes **8**,**9**.

Covalently linked biscalix[5]arenes **8** and **9** produce a preorganized cavity between the two calix[5]arene moieties (Figure 4a). These biscalix[5]arenes encircle the fullerene spheres, maximizing intermolecular interactions. Synthesis of biscalix[5]arenes **8** and **9** involved Sonogashira and Glaser coupling of acetyl‐protected calix[5]arenes **10** and **11**, respectively, followed by deprotection of the acetyl groups (Figure 4b) [69]. The shape and dimensions of the biscalix[5]arene cavities were customized to match the shape of fullerene molecules. Binding constants between biscalix[5]arenes **8** and **9** and [60]fullerene were reported as 9,000 and 68,000 L mol^–1^, respectively (Table 2) [65, 70, 71]. These values exceed those estimated for calix[5]arenes **4**–**6** and [60]fullerene (Table 1), indicating efficient stabilization of a [60]fullerene molecule by the surrounding calix[5]arene moieties. Higher binding constants were observed between biscalix[5]arenes **8** and **9** and [70]fullerene compared to [60]fullerene (Table 2). Although the position of the [70]fullerene molecule relative to the biscalix[5]arene cavity has not been determined [69], the [70]fullerene molecule is well stabilized by the surrounding calix[5]arene moieties. These reports highlight the fact that appropriately designed, tailored cavities provide very high binding affinity for the desired guest molecules. Although biscalix[5]arene successfully stabilizes the host–guest complex with fullerenes, its high binding affinity inherently limits the release of fullerenes from the cavity in a controllable manner.

**TABLE 2:** Summary of binding constants (*K*_a_/L mol^–1^) for interactions between calix[5]arenes **8,9** and [60]/[70]fullerenes in toluene.

	C_60_	C_70_
**8**	9,000 ± 200	91,000 ± 9,000
**9**	68,000 ± 5,000	118,000 ± 12,000

**FIGURE 5:**
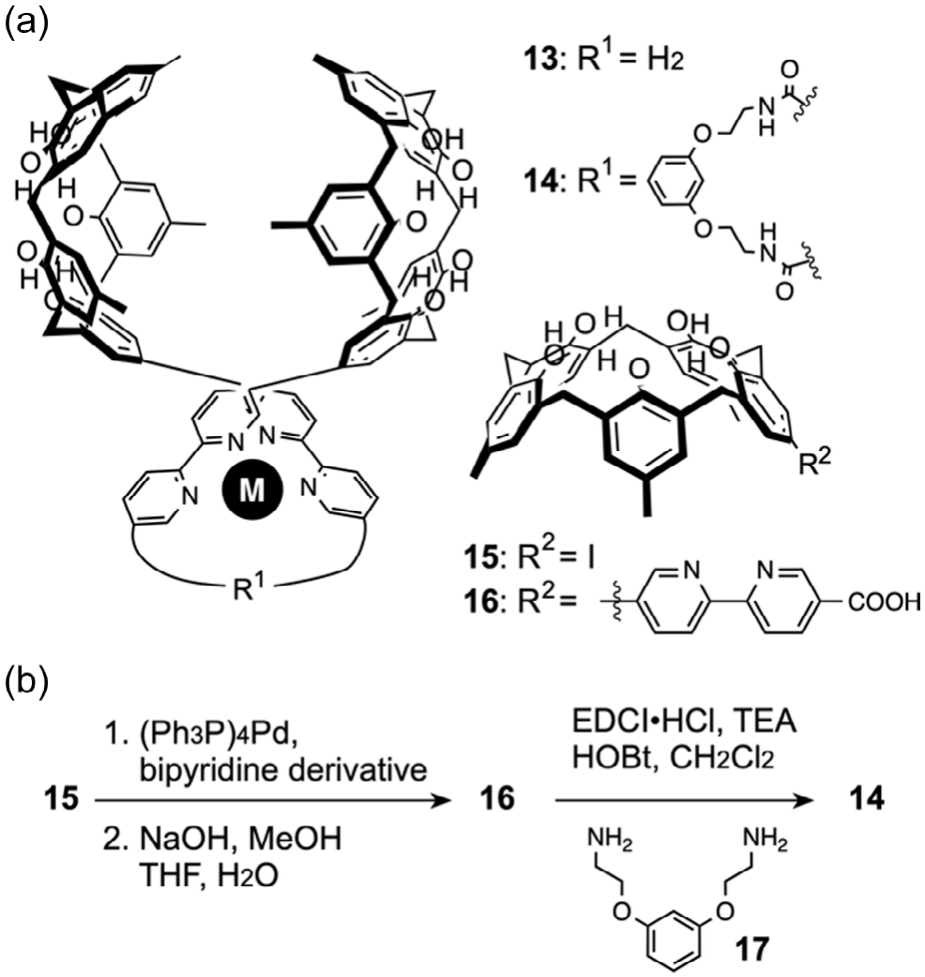
(a) Molecular structure of bipyridine‐appended calix[5]arenes **13**,**14**. (b) Synthesis of bipyridine‐appended calix[5]arene **14**.

To regulate the encapsulation–release process, we introduced a bipyridine ligand into the calix[5]arene structure. Bipyridine‐appended calix[5]arenes **13**,**14** (Figure 5a) were synthesized following the procedure depicted in Figure 5b [72, 73]. Palladium‐mediated coupling of calix[5]arene monoiodide **15** with (trimethylstannyl)bipyridine, followed by hydrolysis, produced bipyridine‐appended calix[5]arenes **13** and **16**. Subsequent dehydration condensation of the carboxyl group of **16** with diamine **17** led to the formation of calix[5]arene **14**. Biscalix[5]arene **13**•Ag^+^•**13** was obtained by simply adding Ag^+^ to a solution of **13**. As shown in Figure 5a, a biscalix[5]arene cavity was defined within the molecular structure of **14** when the bipyridine moieties formed the dative bonds with Cu^+^. Calix[5]arenes **13**,**14** create a cavity between two calix[5]arene units, and the biscalix[5]arene cavity is activated solely in the presence of metal cations (Figure 5a). The [60]fullerene molecule exhibits greater suitability for the biscalix[5]arene cavity compared with the calix[5]arene cavity, allowing the modulation of the binding affinity between calix[5]arenes **13**,**14** and [60]fullerene by introducing metal cations. As summarized in Table 3, the binding constants between **14** and [60]/[70]fullerenes were 90 and 250 L mol^–1^, respectively. Notably, higher binding constants of 3,800 L mol^–1^ for [60]fullerene and 950 L mol^–1^ for [70]fullerene were achieved when **14** interacted with the fullerenes in the presence of Cu^+^. The two calix[5]arene units of **14** exhibit free rotation in solution due to their flexible linker moiety. Upon encapsulation of [60]fullerene by one calix[5]arene unit of **14**, the other unit wraps around the opposite side of [60]fullerene to optimize intermolecular interactions between **14** and [60]fullerene. This process restricts the conformational freedom of **14**, leading to a reduction in entropy. In contrast, conformational freedom of metal‐coordinated **14** (**14**•Cu^+^•**14**) remains largely unchanged before and after encapsulating [60]fullerene due to the fixed orientation of the two calix[5]arene units by the bipyridine moieties’ dative bonds. Therefore, **14** in the absence of Cu^+^ resulted in a lower binding affinity between the fullerenes than that between **14** and the fullerenes in the presence of Cu^+^. The activation and deactivation of the biscalix[5]arene cavities of **13** and **14** are reversible through coordination with metal cations; accordingly, **13** and **14** act as stimulus‐responsive host molecules. This is an example of the judicious use of the difference in the binding affinities of calix[5]arenes and biscalix[5]arenes.

**TABLE 3:** Summary of binding constants (*K*_a_/L mol^–1^) for the interactions between calix[5]arenes **14** and [60]/[70]fullerenes in (CHCl_2_)_2_, with and without Cu^+^.

	C_60_	C_70_
**14**	98 ± 2	250 ± 20
**14** + Cu	3,800 ± 300	950 ± 50

**FIGURE 6:**
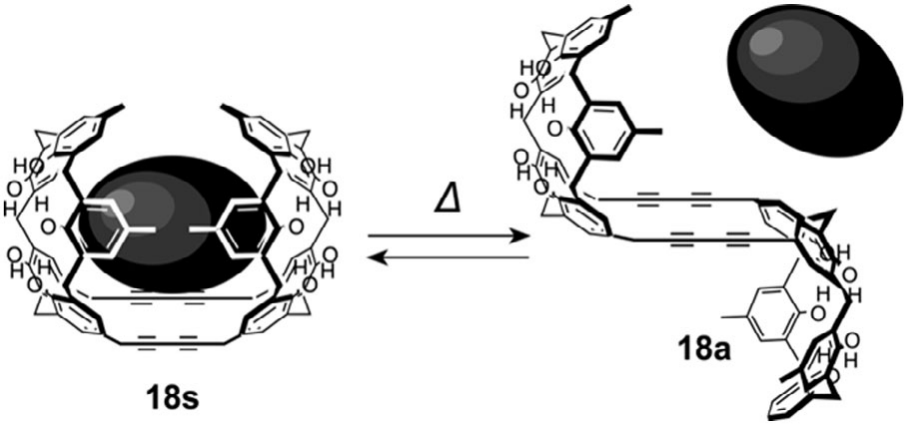
Schematic illustration of the encapsulation and release of fullerene through conformational interconversion of biscalix[5]arene **18**.

Doubly bridged biscalix[5]arenes create more rigidly preorganized cavities compared to singly bridged biscalix[5]arenes (e.g., **8** and **9**), reducing the entropic penalty linked to complexation. This construction enables the formation of fullerene host–guest complexes with exceptionally high binding affinities. To further enhance fullerene encapsulation affinity, a conformationally rigid cavity was designed by linking two calix[5]arene units with two butadiyne linkers to produce biscalix[5]arene **18** (Figure 6) [74]. Biscalix[5]arene **18** exists in two atropisomers: a syn‐form (**18s**), where the two calix[5]arene units face each other to create a well‐defined cavity, and an anti‐form (**18a**), where the two calix[5]arene units are oriented outward. **18s** exhibited significantly enhanced binding constants for [60]fullerene and [70]fullerene compared to singly bridged biscalix[5]arene host **8** in (CHCl_2_)_2_ (Table 4), owing to the conformationally rigid preorganized cavity of **18s**, offering a notable entropic advantage. Importantly, the syn–anti isomerization of **18** was thermally controllable, facilitating the development of a practical extraction method for higher fullerenes from fullerene mixtures containing [60]fullerene (67%) and [70]fullerene (23%). Upon mixing **18s** with the fullerene mixture in toluene, selective precipitation of the host–guest complexes between **18s** and higher fullerenes was observed. Subsequent heating induced the syn‐to‐anti transformation of **18**, leading to the release of the encapsulated higher fullerenes. This process was iterated to enrich the higher fullerenes, effectively excluding the [60]fullerene in the final extract. These findings illustrate that conformationally switchable biscalix[5]arenes offer a robust strategy for the selective encapsulation and release of fullerenes through thermal input.

**TABLE 4:** Summary of binding constants (*K*_a_/L mol^–1^) for the interactions of calix[5]arenes **8,18s** with fullerenes in (CHCl_2_)_2_.

	C_60_	C_70_	C_76_	C_78_	C_84_
**8**	1,550 ± 40	2,800 ± 100	1,850 ± 70	2,500 ± 200	110 ± 10
**18s**	9,700 ± 100	29,000 ± 2,000	90,000 ± 10,000	110,000 ± 20,000	20,000 ± 2,000

## 3 Multitopic Biscalix[5]arene Hosts

**FIGURE 7:**
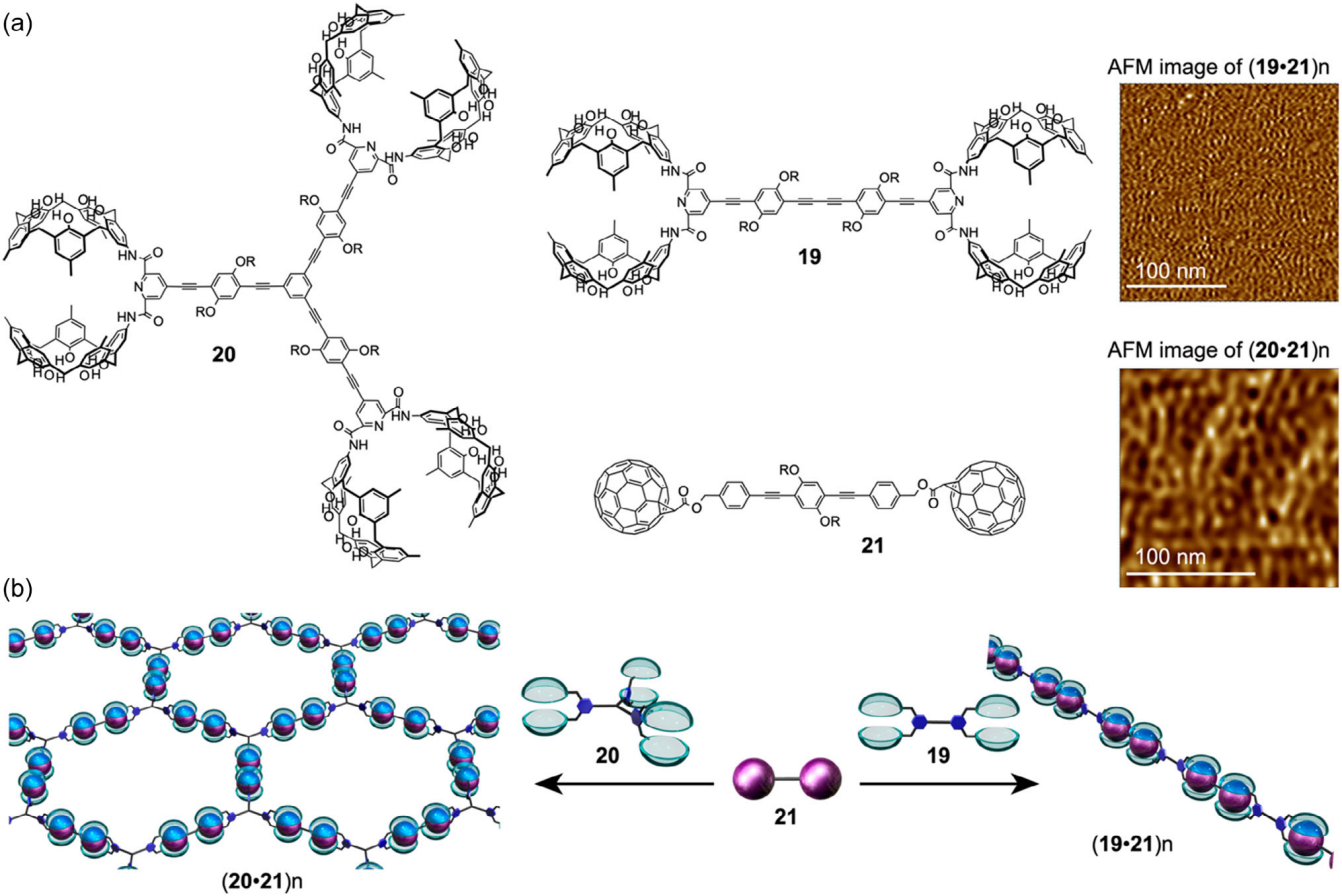
(a) Molecular structures of multi(biscalix[5]arene)‐tethered hosts **19** and **20**, and dumbbell‐shaped fullerene **21**. (b) Schematic illustration of the formation of linear and netting supramolecular fullerene polymers via the host–guest complexation between biscalix[5]arene and [60]fullerene. Reproduced with permission. [89] Copyright 2014, Wiley‐VCH.

Multi(biscalix[5]arene)‐tethered hosts extend the calix[5]arene–fullerene host–guest chemistry from discrete assemblies to polymeric structures. These noncovalently linked polymers are classified as supramolecular polymers, representing a novel class of polymer [75, 76, 77, 78, 79, 80, 81, 82]. Owing to the reversible nature of noncovalent interactions, supramolecular polymers exhibit inherent stimulus‐responsive properties, attracting significant interest in functional polymer materials [83, 84, 85, 86, 87, 88]. The biscalix[5]arene)‐tethered hosts encapsulate the [60]fullerene moieties of multi(fullerene)‐tethered guests iteratively, resulting in a regular array of fullerenes along the supramolecular polymer backbone. We successfully synthesized ditopic and tritopic biscalix[5]arene host molecules **19** and **20**, along with dumbbell‐shaped fullerene **21** (Figure 7a) [89, 90]. The formation of biscalix[5]arene–[60]fullerene host–guest complexes between multitopic hosts **19**,**20** and dumbbell‐shaped fullerenes **21** in solution was meticulously examined using ^1^H nuclear magnetic resonance (NMR), ultraviolet/visible (UV/Vis) absorption, and fluorescence measurements. Diffusion‐ordered NMR spectroscopy (DOSY) is a valuable method for estimating the degree of polymerization (DP) in solution. By measuring the diffusion coefficients (*D*) of molecular species, DOSY provides data convertible into hydrodynamic radii (*r*_h_) using the Stokes–Einstein equation: *D* = *k*_B_ *T*/6*πηr*_h_, where *k*_B_ is the Boltzmann constant, *η* is the solvent viscosity, and *T* represents the temperature. This relationship allows a rough estimation of the DP at each concentration. Based on the measured *D* values, molecular assemblies in the solutions of mixtures **19** and **21**, and **20** and **21** corresponded to (**19**•**21**)_*n*_ and (**20**•**21**)_*n*_ with DPs of 77 and 130, respectively, at a concentration of 11 mmol L^–1^. Furthermore, the solution viscosity of the **20** and **21** mixture increased more significantly than that of the **19** and **21** mixture, indicating effective interchain contacts from the network structures. This behavior suggests the formation of linear and netting fullerene polymers in the solution (Figure 7b). Here, we must note that the present DP values were derived under the assumption of hard spheres. Thus, although the relative changes in its magnitude can be discussed, the absolute values of DP should be regarded as no more than rough approximations. Atomic force microscopy (AFM) was employed to visualize 1D fibrous morphologies and honeycomb‐patterned network morphologies on mica for mixtures of **19** and **21** and **20** and **21**, respectively. These observations underscore that purposefully designed host molecules can be utilized to regulate supramolecular polymer morphology.

**FIGURE 8:**
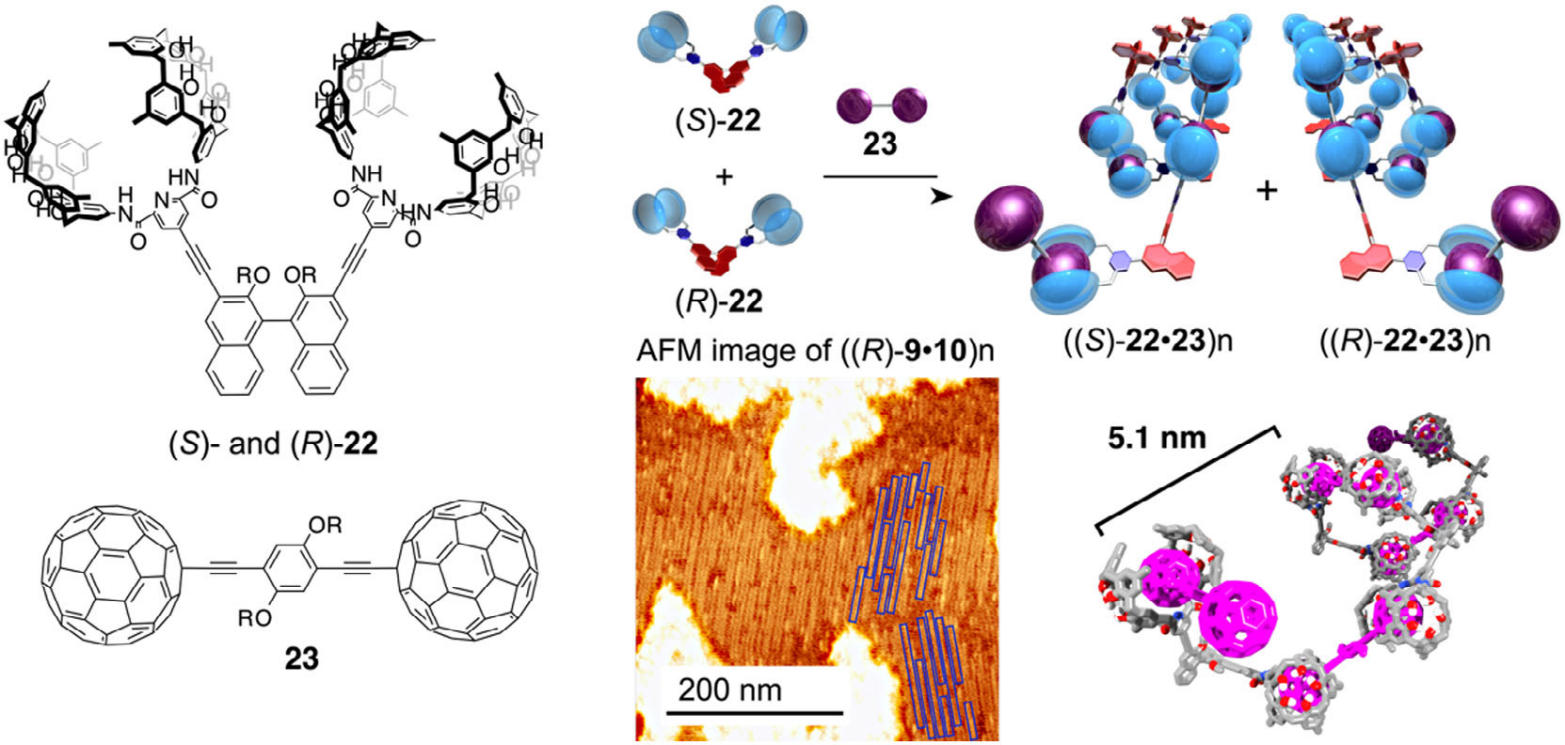
Molecular structures of chiral ditopic biscalix[5]arene hosts (*R*)‐, (S)‐**22**, and dumbbell‐shaped fullerene **23**, along with a schematic illustration depicting the formation of helical supramolecular fullerene polymers. Reproduced with permission. [91] Copyright 2021, ACS.

Helical structures have expanded the architectural range of supramolecular fullerene polymers. After linear and networked architectures, more advanced higher‐order structures, such as helices and chirality‐regulated sequences, have been demonstrated. We successfully created a helically organized fullerene array within a supramolecular polymer main chain (Figure 8) [91]. Two biscalix[5]arene units were covalently linked to a nonracemic binaphthoxy group, inducing a chirally twisted spatial arrangement on the biscalix[5]arene moieties. The ditopic chiral hosts (*R*)‐ and (*S*)‐**22** formed calix[5]arene–[60]fullerene host–guest complexes iteratively, resulting in helical supramolecular fullerene polymers ((*R*)‐**22**•**23**)_*n*_ and ((*S*)‐**22**•**23**)_*n*_, where the chirality of the binaphthoxy linkers determined the helical direction of the polymer backbone. Circular dichroism (CD) measurements showed bisignate bands from the fullerene moieties, confirming that achiral fullerene units were twisted into chiral conformations through supramolecular polymerization. The helical structure of ((*R*)‐**22**•**23**)_*n*_ and ((*S*)‐**22**•**23**)_*n*_ reduces its conformational flexibility and gives rise to a rod‐like shape, which should be regarded as a deviation from the hard‐sphere model. A modified Stokes–Einstein equation, the so‐called cylindrical model, has been reported for cylindrical molecular species. The helical structure of ((*R*)‐**22**•**23**)_*n*_ and ((*S*)‐**22**•**23**)_*n*_ can be reasonably approximated by a cylinder, and the corresponding cylindrical model is given by *D* = (*k*_B_ *T*/3*πηr*_h_) (ln*p* + 0.312 + 0.565*p* ^–1^ – 01.00*p* ^–2^) [92, 93, 94, 95, 96, 97], where *p* is the axial ratio (*L*/*d*; *L* = cylinder length, *d* is the cylinder diameter). The DP of ((*R*)‐**22**•**23**)_*n*_ was evaluated to be 32 at a concentration of 30 mmol L^–1^ on the basis of the cylindrical approximation, whereas a DP of 31 was obtained from the binding constant between biscalix[5]arene and [60]fullerene. Considering the fact that DP of 204 was estimated for ((*R*)‐**22**•**23**)_*n*_ at a concentration of 30 mmol L^–1^ from the hard‐sphere model, rod‐like polymers lead to a substantial overestimation of the actual DP by applying the hard‐sphere model. Therefore, careful model selection is crucial for reliable DP estimation. AFM revealed distinct twisted chains with a consistent pitch of approximately 5 nm, offering direct evidence of helically arranged fullerene arrays within the polymer backbone in the solid state.

A subsequent investigation of the chiral host indicated that (**22**•**23**)_*n*_ displayed notable self‐sorting behavior [98]. When racemic mixtures of (*R*)‐ and (*S*)‐**22** ((*rac*)‐**22**) were mixed with **23**, the homochiral supramolecular polymers were predominantly produced. The DOSY measurements revealed a significantly reduced DP in the (*rac*)‐**22** and **23** mixture compared with that in the (*R*)‐**22** and **23** mixture, suggesting the distinct formation of ((*R*)‐**22**•**23**)_*n*_ and ((*S*)‐**22**•**23**)_*n*_. CD spectroscopy validated the preferential formation of right‐ and left‐handed helices, illustrating that supramolecular polymerization encodes stereoregularity through self‐sorting.

**FIGURE 9:**
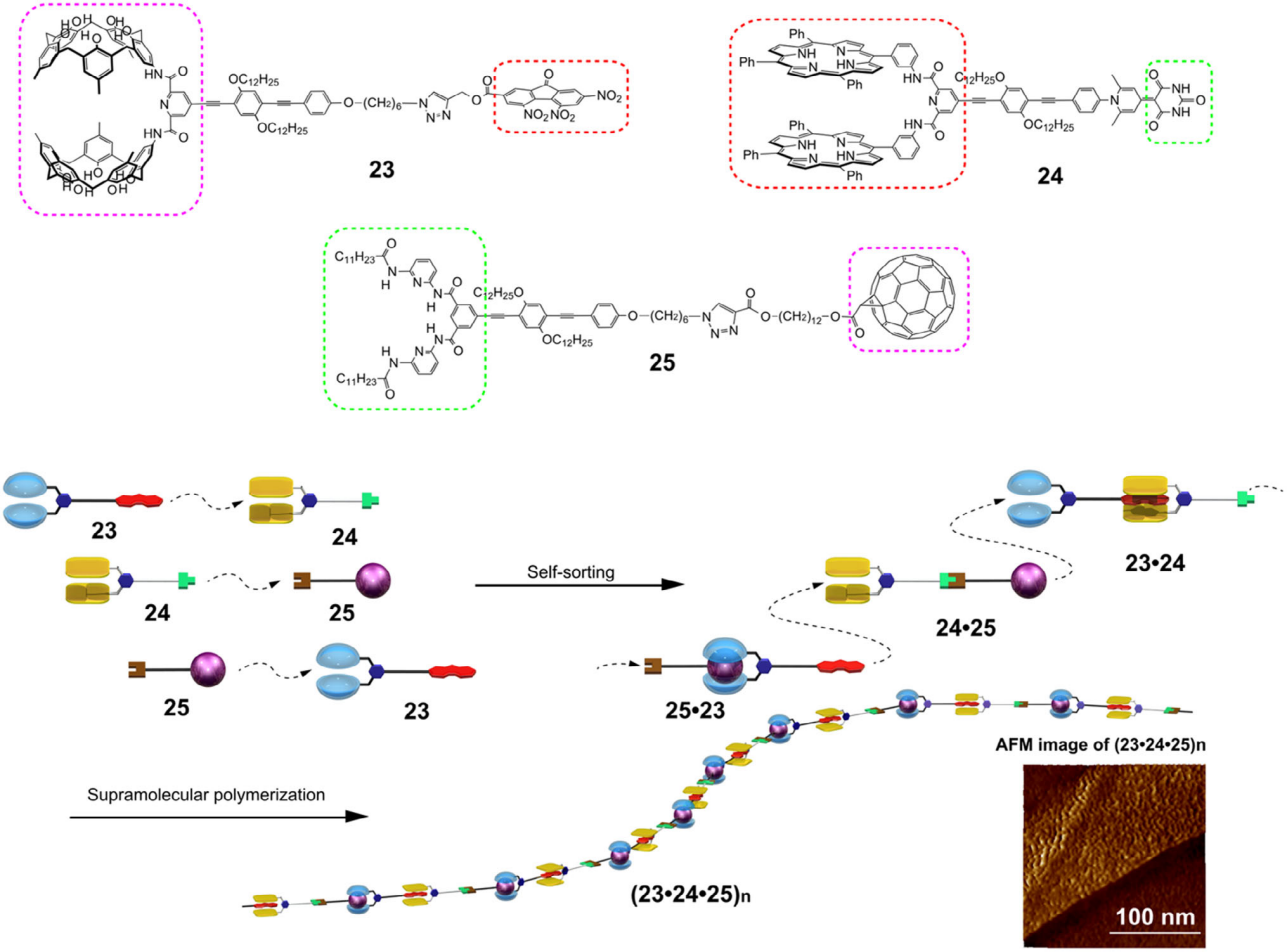
Molecular structures of host‐ and guest‐appended monomers **23**, **24**, and **25** and a schematic illustration of the formation of linear supramolecular polymers with a repeated array of **23**, **24**, and **25**. Reproduced with permission. [99] Copyright 2017, NPG.

In addition to its helical organization, sequence regulation offers another dimension to broaden the architectural scope of supramolecular fullerene polymers. Inspired by the precisely controlled monomer sequence in biopolymers, we demonstrated a sequence‐controlled supramolecular terpolymerization guided by self‐sorting [99]. In this approach, three orthogonal host–guest pairs served as building blocks for the terpolymer: biscalix[5]arene–[60]fullerene, bis(porphyrin)–trinitrofluorenone, and Hamilton's bis(acetamidopyridinyl)isophthalamide–barbiturate hydrogen bonding. These complementary interactions were integrated into heteroditopic monomers that selectively recognized each other, thereby facilitating the intermolecular head‐to‐tail assembly of the supramolecular terpolymer (**23**•**24**•**25**)_*n*_ (Figure 9).

^1^H NMR and UV/Vis titrations confirmed the selective formation of heterodimers (**23**•**24**, **24**•**25**, and **25**•**23**). ESI‐MS detected oligomeric fragments consistent with the repeating **23–24**–**25** sequence, excluding scrambled products. DOSY and viscometry revealed large supramolecular polymers in solution. AFM images of an equimolar mixture of **23**, **24**, and **25** displayed fibrous morphologies, while random aggregates were observed in AFM images of **23**, **24**, and **25**; a mix of **23** and **24**; a mix of **24** and **25**; and a mix of **25** and **23**, indicating the formation of (**23**•**24**•**25**)_*n*_ in the solid state.

This study shows that the calix[5]arene–fullerene host–guest complex can be effectively integrated with additional complementary interactions, providing a basis for creating customized supramolecular polymers with sequence‐encoded functionalities. These sequence‐regulated supramolecular polymers are anticipated to pave the way for advancing information‐rich polymeric materials.

## 4 Biscalix[5]arene Cavities for Polymer Functionalization

**FIGURE 10:**
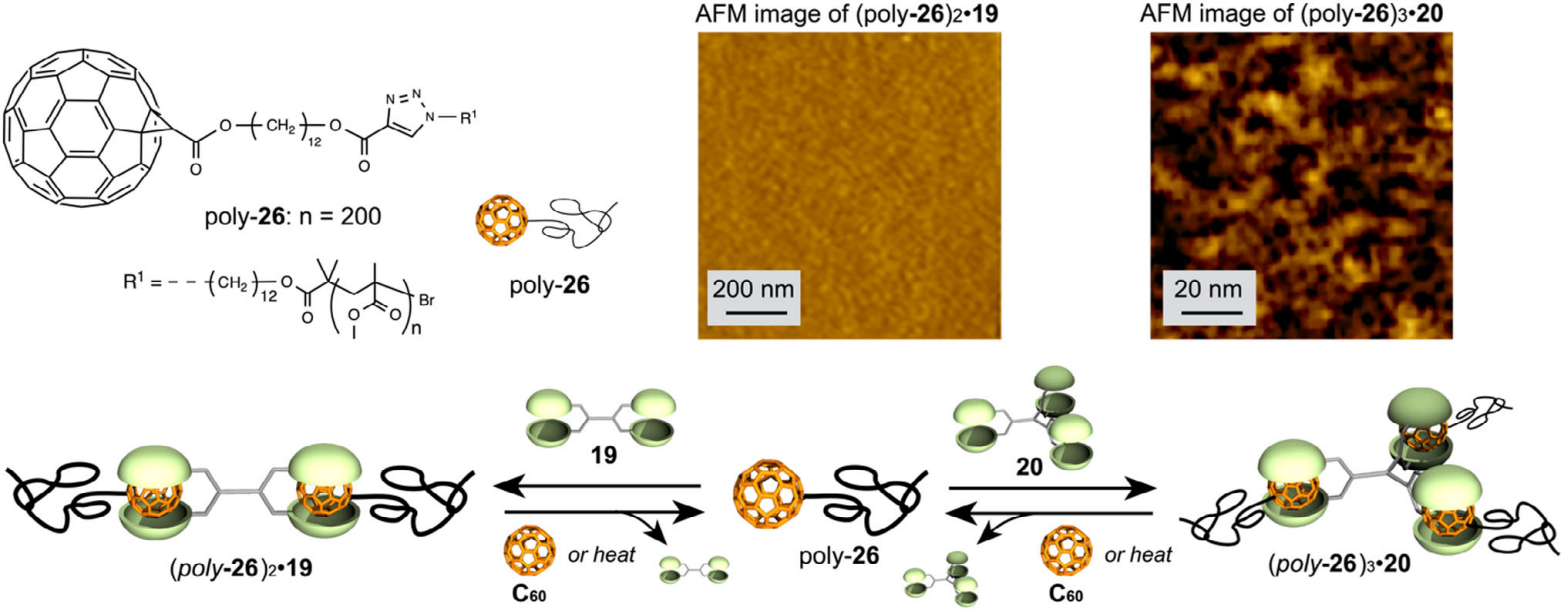
Molecular structure of [60]fullerene‐appended polymethyl methacrylates (PMMA) poly‐**26**, and schematic illustration of the formation of linear and star PMMA via host–guest complexation. Reproduced with permission. [104] Copyright 2020, ACS.

The integration of host–guest interactions into polymer chains has become a potent method for introducing dynamic behavior to macromolecular architectures [64, 100, 101, 102, 103]. The strong affinity between biscalix[5]arenes and fullerenes allows for connecting polymer chains, resulting in uniquely shaped polymers. We utilized calix[5]arene–fullerene host–guest interactions to showcase a supramolecular strategy for altering polymer shapes using fullerene‐appended polymethyl methacrylates (PMMAs) (Figure 10) [104]. Linear ditopic and branched tritopic biscalix[5]arene hosts **19** and **20** selectively encapsulated the [60]fullerene unit at the chain termini of PMMA. UV/Vis and fluorescence quenching analyses confirmed the formation of calixarene–fullerene complexes, while DOSY and size‐exclusion chromatography (SEC) indicated an increase in hydrodynamic radii when poly‐**26** was combined with **19** and **20**, suggesting chain elongation and branching. Viscometry and differential scanning calorimetry supported the creation of distinct polymer shapes, specifically (poly‐**26**)_2_•**19** and (poly‐**26**)_3_•**20**, by demonstrating viscosity alterations and modified glass transition temperatures (*T*_g_) compared with poly‐**26** without the hosts. AFM revealed smooth amorphous‐like morphologies on the substrate, consistent with the topographic PMMA images.

Notably, (poly‐**26**)_2_•**19** and (poly‐**26**)_3_•**20** exhibited stimuli‐responsive shape interconversion. Dynamic light scattering revealed reversible switching between short, linear, and star‐shaped PMMAs during heating–cooling cycles or competitive binding with pristine [60]fullerene, displacing PMMA from the biscalix[5]arene cavity. This study highlights that biscalix[5]arene cavities can link polymer chains and encode shape plasticity, providing a powerful tool for developing stimuli‐responsive, multistate polymeric materials.

**FIGURE 11:**
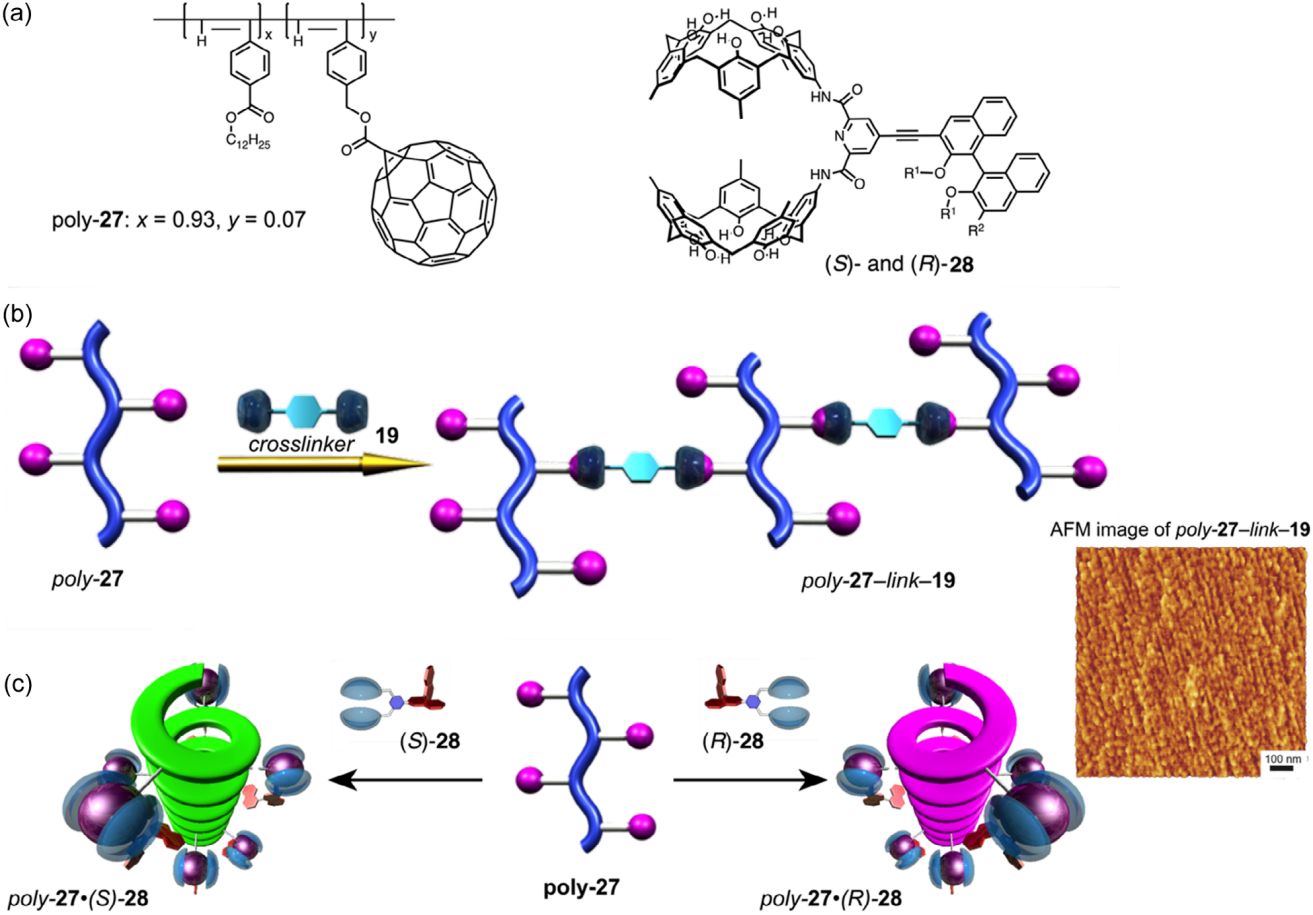
(a) Molecular structure of [60]fullerene‐appended polyphenylacetylene (PPA) derivatives poly‐**27a**,**b**, (*S*)‐**28**, and (*R*)‐**28**. Schematic illustration of the formation of (b) cross‐linked polystyrene (PS) and (c) one‐handed helical PPA. Reproduced with permission from Ref. [105] Copyright 2010, Wiley‐VCH.

The homoditopic host **19** serves as a fundamental component in the formation of cross‐linked polymers by incorporating two or more fullerene moieties into the polymer chains [105]. We report the successful synthesis of [60]fullerene‐appended polyphenylacetylene (PPA) poly‐**27,** which was cross‐linked with **19** through calix[5]arene–[60]fullerene host–guest complexation (Figure 11a,b). ^1^H NMR and fluorescence quenching experiments established that the calix[5]arene cavities selectively encapsulate the [60]fullerene moieties in the PPA side chains. SEC revealed a significant increase in the molecular weight of poly‐**27** upon the addition of **19**, confirming the formation of stable supramolecular crosslinked polymers. Support for the formation of cross‐linked PPA in the solid state was obtained from the AFM measurements. Poly‐**27** aggregated into nanoparticle‐like morphologies on the substrates, whereas well‐oriented fibrous networks were visualized in the AFM image of a mixture of poly‐**27** and **19**, indicating structural reorganization through host–guest complexation.

A decade later, we expanded the host–guest concept between PPA and biscalix[5]arenes to regulate the helix sense of PPA main chains [106]. Chiral binaphthyl‐based monotopic biscalix[5]arene hosts (*R*)‐**28** and (*S*)‐**28**, whose chirality arises from the binaphthyl unit, were synthesized (Figure 11a) and combined with poly‐**27** to create one‐handed helical polymers (Figure 11c). CD spectroscopy demonstrated a mirror‐image relationship for the CD bands of poly‐**27** when (*R*)‐ and (*S*)‐**28** were present, indicating induced one‐handed helicity in the PPA main chain, with the preferred helix sense corresponding to the (*R*) and (*S*) chiralities of **28**. The helical sense of the PPA main chain is believed to be transmitted from the stereogenic axis of the binaphthyl moiety of **28** to the PPA main chain through the calix[5]arene–fullerene interface. Force‐field calculations supported this hypothesis, showing that the direct interaction between the binaphthyl unit and the biscalix[5]arene moiety causes a slight twist of the two calix[5]arene cavities in a chiral manner. This twist provides steric communication that is effectively conveyed to the polymer main chain, inducing the preferred helicity in the PPA main chain.

**FIGURE 12:**
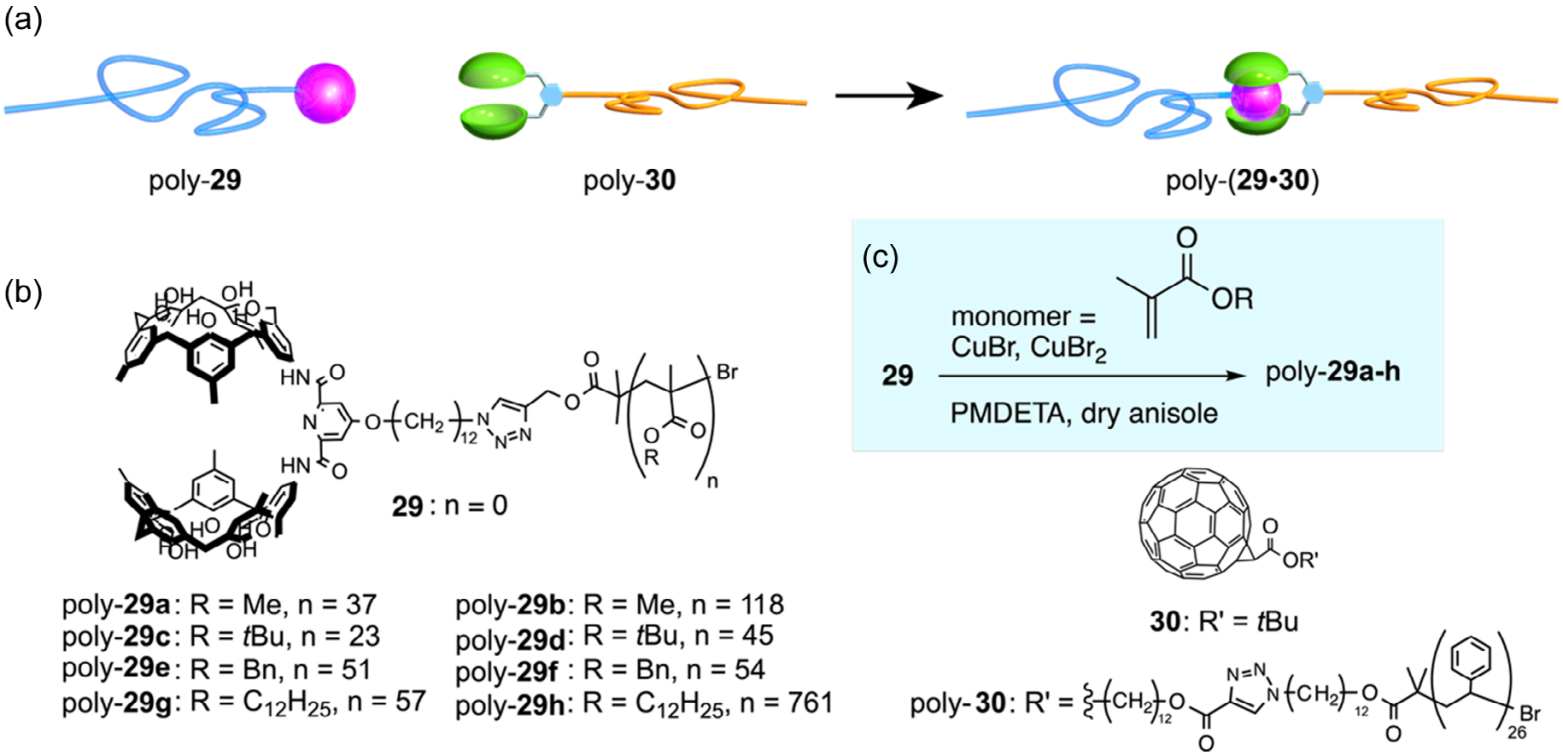
(a) Schematic illustration of the formation of a block polymer via the calix[5]arene–[60]fullerene host–guest complexation. (b) Molecular structure of polyalkylmethacrylates poly‐**29a**–**h** and [60]fullerene‐appended PS poly‐**30**. (c) Synthesis of poly‐**29**. Reproduced with permission from Ref. [107] Copyright 2021, RSC.

Biscalix[5]arene cavities have also been used to construct block copolymers (Figure 12a,b) [107]. Our group demonstrated that biscalix[5]arene‐appended methacrylate monomer **29** tolerates atom transfer radical polymerization (ATRP), affording a series of biscalix[5]arene‐functionalized polymethacrylates (poly‐**29a**–**h**) with different side chains (Figure 12c). Independent of the alkyl substituent group of the polymethacrylate chains, the biscalix[5]arene moiety of poly‐**29** displayed a binding constant on the order of 10^4^ L mol^–1^ with a [60]fullerene derivative **30**, as summarized in Table 5. This observation indicates that poly‐**29** retains the ability to form stable calix[5]arene–fullerene host–guest complexes, even when long polymer chains are present.

**TABLE 5:** Summary of binding constants (*K*_a_/L mol^–1^) for interactions between poly‐**29a–h** and **30** in chloroform.

	poly‐29a	poly‐29b	poly‐29c	poly‐29d	poly‐29e	poly‐29f	poly‐29g	poly‐29h
*K*_a_	26,000 ± 1,000	15,000 ± 1,000	17,000 ± 300	15,300 ± 600	15,000 ± 500	13,000 ± 1,000	20,500 ± 400	19,700 ± 700

Mixing poly‐**29c** with poly‐**30** resulted in the formation of a supramolecular block copolymer through biscalix[5]arene–fullerene complexation, as confirmed by UV/Vis titration experiments. This outcome, demonstrated with a single polymer pair, highlights the potential of biscalix[5]arene–fullerene host–guest complexation as a reliable and versatile non‐covalent junction for constructing supramolecular block copolymers. These discoveries enhance the functionality of biscalix[5]arene cavities, facilitating stimuli‐responsive polymer transformations to act as efficient connectors for block polymers and expanding the range of supramolecular polymer designs.

## 5 Summary and Outlook

In this review, we outline the design principles for molecular cavities utilizing calix[5]arenes and their supramolecular polymeric extensions, referencing our reports. The introduction briefly discusses the importance of cavity design for selective guest encapsulation, citing key contributions in this field. Subsequent sections detail our efforts in customizing calix[5]arene‐based hosts, emphasizing the binding affinities determined by the shape and dimensions of their cavities. We also present our versatile hosts for supramolecular fullerene polymers and shape‐modifiable polymers. Finally, a biscalix[5]arene–[60]fullerene host–guest complexation as an interpolymer chain connector is described.

Artificial host–guest complexation was first investigated in the late 1950s [108], and the discovery of crown ether in 1967 marked a turning point, leading to the establishment of “host–guest chemistry.” [109, 110] Since then, host–guest chemistry has undergone rapid development, driven by significant contributions from numerous research groups worldwide [111, 112, 113, 114, 115, 116, 117, 118, 119, 120, 121, 122, 123, 124, 125, 126, 127, 128, 129, 130, 131, 132, 133, 134, 135, 136]. The subsequent development in the field have underscored the importance of rational cavity design as a fundamental principle underlying host–guest complexation. In this context, host–guest chemistry is closely related to the design and tailoring of cavities to achieve the desired shape and dimensions. In other words, host–guest chemistry focuses on the rational design of cavities to accommodate target guest molecules. Advances in host–guest chemistry have been driven by the designable features of synthetic host molecules. The designable features of synthetic host molecules allow us to tailor the shapes and dimensions of their cavities to make them suitable for the target molecules. Thus, judiciously tailored cavities encapsulate ideal partner guests in a selective manner to form host–guest complexes, leading to various applications, as briefly mentioned in the Introduction. Furthermore, well‐designed specific host–guest complexes have been used as building blocks for more complicated and well‐organized molecular assemblies [137, 138, 139, 140, 141, 142, 143, 144]. In recent years, our host–guest chemistry has been extended to nonmacrocyclic host molecules [145]. This development highlights that host–guest chemistry can be advanced beyond conventional cavity designs based on cyclic frameworks by creating intermolecular cavities using non‐macrocyclic molecules. As such, beyond their intrinsic aesthetic appeal [146], host–guest complexes are promising building blocks in the development of well‐organized molecular assemblies, as their assembled structures can be tailored by adjusting the design of the host–guest complexes. We hope that our work will contribute to the continued evolution of host–guest chemistry.

## Conflicts of Interest

The authors declare no conflicts of interest.

## Data Availability

Data sharing is not applicable to this article as no new data were created or analyzed in this study.
